# Quasielastic neutron scattering study on proton dynamics assisted by water and ammonia molecules confined in MIL-53

**DOI:** 10.1063/4.0000122

**Published:** 2021-10-12

**Authors:** Satoshi Miyatsu, Maiko Kofu, Akihito Shigematsu, Teppei Yamada, Hiroshi Kitagawa, Wiebke Lohstroh, Giovanna Simeoni, Madhusudan Tyagi, Osamu Yamamuro

**Affiliations:** 1 Institute for Solid State Physics, University of Tokyo, 5-1-5 Kashiwanoha, Kashiwa, Chiba 277-8581, Japan; 2 Division of Chemistry, Graduate School of Science, Kyoto University, Kitashirakawa Oiwake-cho, Sakyo-ku, Kyoto 606-8502, Japan; 3 Heinz Maier-Leibnitz Zentrum (MLZ), Technische Universität München, D-85747 Garching, Germany; 4 NIST Center for Neutron Research, National Institute of Standards and Technology, 100 Bureau Drive, Gaithersburg, Maryland 20899-6102, USA; 5 Department of Materials Science, University of Maryland, College Park, Maryland 20742, USA

## Abstract

Dynamics of water and other small molecules confined in nanoporous materials is one of the current topics in condensed matter physics. One popular host material is a benzenedicarboxylate-bridging metal (III) complex abbreviated to MIL-53, whose chemical formula is M(OH)[C_6_H_2_(CO_2_)_2_R_2_] where M = Cr, Al, Fe and R = H, OH, NH_2_, COOH. These materials absorb not only water but also ammonia molecules. We have measured the quasi-elastic neutron scattering of MIL-53(Fe)-(COOH)_2_·2H_2_O and MIL-53(Fe)-(COOH)_2_·3NH_3_ which have full guest occupancy and exhibit the highest proton conductivity in the MIL-53 family. In a wide relaxation time region (*τ* = 10^−12^–10^−8^ s), two relaxations with Arrhenius temperature dependence were found in each sample. It is of interest that their activation energies are smaller than those of bulk H_2_O and NH_3_ liquids. The momentum transfer dependence of the relaxation time and the temperature dependence of the relaxation intensity suggest that the proton conduction is due to the Grotthuss mechanism with thermally excited H_2_O and NH_3_ molecules.

## I. INTRODUCTION

Metal–organic frameworks (MOFs) have attracted much attention from not only basic scientific interest but also their applications in gas sorption,^1–5^ catalysis,^6–10^ and ionic conductivity.^11–21^ They are composed of metal ions and bridging organic ligands to construct porous structures. There are various types of pores, e.g., rectangular cavities, channels, planer gaps, etc. Various small molecules, e.g., water, alcohols, hydrocarbons, are accommodated in the pores. The geometry, size, and surface condition (hydrophilic, hydrophobic, etc.) of pores can be controlled by changing metal ions and ligands.^22–27^

We have focused on MOFs that exhibit higher proton conductivity. High-performance proton conductors are desirable in fuel cell technology.^28–33^ Protons are supplied by groups such as COOH, OH, NH on the pore wall and are carried by guest molecules inside the pores.^34–38^ Water is the most popular carrier. For example, in copper rubeanate (H_2_C_2_N_2_S_2_Cu; H_2_dtoaCu),^39–41^ the proton conductivity is mainly governed by the protons provided by the NH group on the pore wall. H2dtoaCu adsorbs water in its pores to maximum hydration of 3.7 molecules at a relative humidity (RH) of 100%. The conductivity at this concentration is 0.01 S cm^−1^, comparable to that of Nafion, the best commercial proton conductor available. Our quasi-elastic neutron scattering (QENS) study has revealed that the water inside the pore behaves as bulk water and serves as the proton carrier.^42,43^ In the case of (NH_4_)_2_(adp)[Zn_2_(ox)_3_]·3H_2_O (adp = adipic acid, ox = oxalate), which also exhibits a proton conductivity as high as 0.01 S cm^−1^, water molecules are not like bulk but located at definite crystallographic positions.^37^ This material has a crystal structure consisting of alternating Zn_2_(ox)_3_ layers and conducting layers which contain COOH groups of adipic acid, H_2_O molecules, and NH_4_^+^ ions. Our QENS work demonstrated that protons are supplied by COOH groups and carried by both H_2_O molecules and NH_4_^+^ ions through the Grotthuss mechanism.^42^

The material taken up in this study is a benzenedicarboxylate-bridging metal (III) complex abbreviated as MIL-53 after the producer institute (Materials Institute of Lavoisier). The chemical formula is M(OH)[bdc-R_2_] where bdc = 1,4-benzene dicarboxylate (O_2_C-C_6_H_2_-CO_2_), M = Cr, Al, Fe and R = H, OH, NH_2_, COOH. These materials absorb not only water but also ammonia molecules.^43^ This may be the first case where NH_3_ molecules potentially carry protons and can be tested. We have taken MIL-53 with M = Fe and R = COOH since it exhibits the highest proton conductivity in MIL-53 systems.^43,44^

**FIG. 1.**
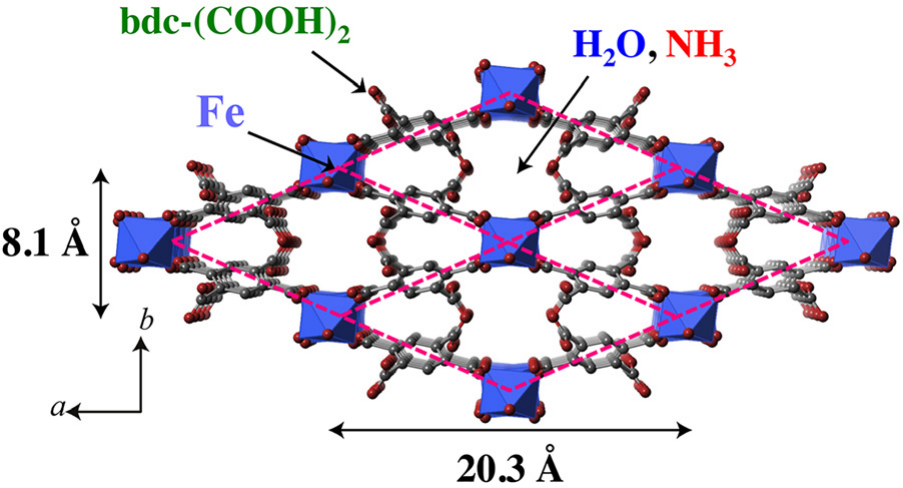
Perspective view along the c-axis of MIL-53(Fe)-(COOH)_2_. Argyle channels, drawn by red lines, are formed by bdc-(COOH)_2_ groups. Fe^3+^ ions are located at the center of octahedrons shaded by blue. Red and gray balls correspond to O and C atoms, respectively (H atoms are omitted). The positions of the H_2_O and NH_3_ molecules in the channels are not known.

Figure 1 shows the crystal structure of MIL-53(Fe)–(COOH)_2_.^45^ This structure has a monoclinic symmetry (space group C2/c); the figure is a view along the c axis. Fe^3+^ ions are coordinated by four bdc and two OH groups to form a framework with argyle channels. For analogous systems without COOH groups (i.e., R = H), the structural transitions with deformation of the argyles were observed at around 200 K.^46,47^ The deformation of the channels (breathing effect) is observed also by gas absorption/desorption processes.^48–50^ In our systems, H_2_O and NH_3_ molecules are accommodated in the argyle channels. Conducting protons may be supplied by COOH groups and carried by H_2_O or NH_3_ molecules. For the full-occupancy samples, MIL-53(Fe)–(COOH)_2_·2H_2_O and MIL-53(Fe)–(COOH)_2_·3NH_3_, proton conductivity is around 10^−6^ and 10^−8^ S cm^−1^, respectively.^43,44^

We have performed the QENS experiments on MIL-53(Fe)–(COOH)_2_·2H_2_O and MIL-53(Fe)–(COOH)_2_·3NH_3_. The purpose of the study is to investigate the motions of the absorbed H_2_O and NH_3_ molecules, and finally to clarify the proton-conducting mechanism. Except the crystal structure and proton conductivity mentioned above, the physical properties of MIL-53(Fe)–(COOH)_2_·2H_2_O and MIL-53(Fe)–(COOH)_2_·3NH_3_ have not been studied so far. It is especially meaningful to clarify the dynamics of the accommodated NH_3_ molecules since the proton transfer via NH_3_ molecules has never been reported before. The QENS experiment for MIL-53 with CO_2_ and light hydrocarbon molecules (methane, ethane, propane, n-butane) have been performed before.^51–53^

## II. EXPERIMENTAL SECTION

### A. Samples preparation

The powder sample of MIL-53(Fe)–(COOH)_2_·*n*H_2_O (*n *≈* *1) was supplied by Kitagawa group. Its synthesis method was described elsewhere.^44^ The sample was kept in a glovebox with a relative humidity of 95% for 6 h to prepare MIL-53(Fe)–(COOH)_2_·2H_2_O. Then the sample was loaded into the double cylindrical Al can (ID of outer can: 14 mmϕ, OD of inner can: 12 mmϕ) for neutron scattering. The thickness of the sample was 1.0 mm, corresponding to a neutron transmission of 93%. This sample was used also for the differential scanning calorimetry (DSC).

The absorption of NH_3_ gas was performed in glass capillary tubes (OD: 1.0 mmϕ, ID: 0.7 mmϕ). First, the original sample was evacuated to remove water in the channels. Complete removal of water was confirmed by a thermogravimetric (TG) method. NH_3_ gas of 0.1 MPa was introduced into the capillary cubes to prepare MIL-53(Fe)–(COOH)_2_·3NH_3_. The stochiometric accommodation of ammonia was confirmed, in advance of the present experiment, by using the elemental analysis, pressure-composition isotherm, and single-crystal x-ray diffraction methods.^43^ Finally, the capillary tubes were sealed by glass melting. For the TOFTOF (Time-of-Flight spectrometer) experiment, 38 capillary tubes were concentrically arranged in the space between the outer can (ID: 22 mmϕ) and inner can (OD: 18 mmϕ). For the HFBS (High-Flux Backscattering Spectrometer) experiment, 45 capillary tubes were arranged in the space between the outer can (ID: 29 mmϕ) and inner can (OD: 26 mmϕ). The neutron transmission was 92% for both experiments.

### B. Sample characterization

The original sample MIL-53(Fe)–(COOH)_2_·*n*H_2_O (*n *≈* *1) and MIL-53(Fe)–(COOH)_2_·3NH_3_ were checked by an x-ray powder diffraction (XRPD) technique. The former sample mounted on a non-reflection Si plate and the latter sample sealed in the capillary tubes were measured with an x-ray powder diffractometer (Rigaku Ultima III, CuKα) in a scattering angle range 5° < 2*θ* < 70°. The diffraction patterns of both samples are essentially the same and the Bragg peak positions are mostly reproduced by the crystal structure of MIL-53(Fe)–(COOH)_2_·0.88H_2_O, which was determined by a previous single-crystal x-ray diffraction experiment.^45^

### C. Differential scanning calorimetry

The thermal properties of MIL-53(Fe)–(COOH)_2_·2H_2_O were measured by a differential scanning calorimeter (Perkin Elmer Diamond DSC). The sample of 4.62 mg was loaded into a seal-type Al pan. The cooling and heating rates were 10 K min^−1^. The DSC experiment of MIL-53(Fe)–(COOH)_2_·3NH_3_ cannot be performed because of the decomposition of the sample during the sample loading.

### D. Quasielastic neutron scattering

The QENS experiments were performed on HFBS^54^ at NIST Center for Neutron Research (NCNR), National Institute of Standards and Technology and TOFTOF^55^ operated by the Technische Universität München and located at Forschungsneutronenquelle Heinz Maier-Leibnitz FRM II.

TOFTOF is a direct-geometry chopper-type spectrometer. The seven rotating disk choppers form the incident neutron pulse with a fixed wavelength. The neutron wavelength, its spread corresponding to the energy resolution, and the pulse repetition can be changed by the chopper conditions. The neutrons are scattered by a sample and detected by 1000 ^3^He tube detectors concentrically located at 4 m from the sample and at a scattering angle range 7° < 2*θ* < 140°. The energy transfer is determined by the time-of-flight method. In the present experiment, the incident neutron wavelength is 9 Å. The corresponding energy transfer range, energy resolution and momentum transfer (*Q*) range are −1 meV < Δ*E *<* *0.6 meV, Δ*E*_res_ = 20 μeV, 0.1 Å^−1^ < *Q *<* *1.3 Å^−1^, respectively. The energy transfer range and resolution roughly correspond to the relaxation time range from 10 to 500 ps.

The fixed window scan (FWS) was performed for both samples from 20 to 300 K at every 10 K with a duration time of 30 min. The QENS data were recorded at 10 K (for resolution) and between 240 and 300 K in 20 K step for MIL-53(Fe)–(COOH)_2_·2H_2_O and between 240 and 320 K in 20 K step for MIL-53(Fe)–(COOH)_2_·3NH_3_. In each QENS run, the counting time was 5 h for MIL-53(Fe)–(COOH)_2_·2H_2_O and 11 h for MIL-53(Fe)–(COOH)_2_·3NH_3_. The neutron powder diffraction (NPD) data were also obtained for both samples using the 1000 detectors with different 2*θ*. For the FWS and NPD, the elastic intensity was determined by integrating the intensity data in an energy range between –10 and 10 *μ*eV. The LAMP software, which was developed by Institut Laue-Langevin (ILL), was used to process the data.^56,57^

HFBS is operated in the dynamic and fixed window modes. In the former mode (QENS measurement conditions), the neutrons, which are diffracted from the rotating phase-space transformer (PST) chopper, are Doppler shifted, providing a neutron wavelength band with its center at 6.27 Å. The neutrons scattered from the sample are energy-analyzed by means of Bragg reflection from Si(111) analyzers at 2.08 meV, and counted on 16 ^3^He detector tubes installed at a scattering angle range 15° < 2*θ* < 120°. The scattering angle at the Si(111) analyzer is 180° (backscattering), minimizing the wavelength spread of analyzed neutrons and realizing the ultra high energy resolution. In this experiment, we used an energy window, −17 *μ*eV < Δ*E *<* *17 *μ*eV, set by the chosen Doppler frequency. The energy resolution was 0.8 *μ*eV, which roughly covers the range of relaxation time from 100 ps to 10 ns. The *Q* range was 0.25 Å^−1^ < *Q *<* *1.75 Å^−1^. In the fixed window mode, the Doppler drive was stopped and only elastic scattering was recorded.

The FWS measurements were performed for both samples in a continuous heating from 6 to 300 K at a rate of 1 K min^−1^. The QENS data were recorded at 7 K (for resolution), 185, 200, 220, 240, and 260 K for MIL-53(Fe)–(COOH)_2_·2H_2_O, and at 220, 240, 260, and 290 K for MIL-53(Fe)–(COOH)_2_·3NH_3_. In each QENS run, the counting time was 8 h for MIL-53(Fe)–(COOH)_2_·2H_2_O and 12 h for MIL-53(Fe)–(COOH)_2_·3NH_3_. The data were processed by the DAVE software developed by NCNR.^58^

## III. RESULTS AND DISCUSSION

### A. Search for a phase transition

**FIG. 2.**
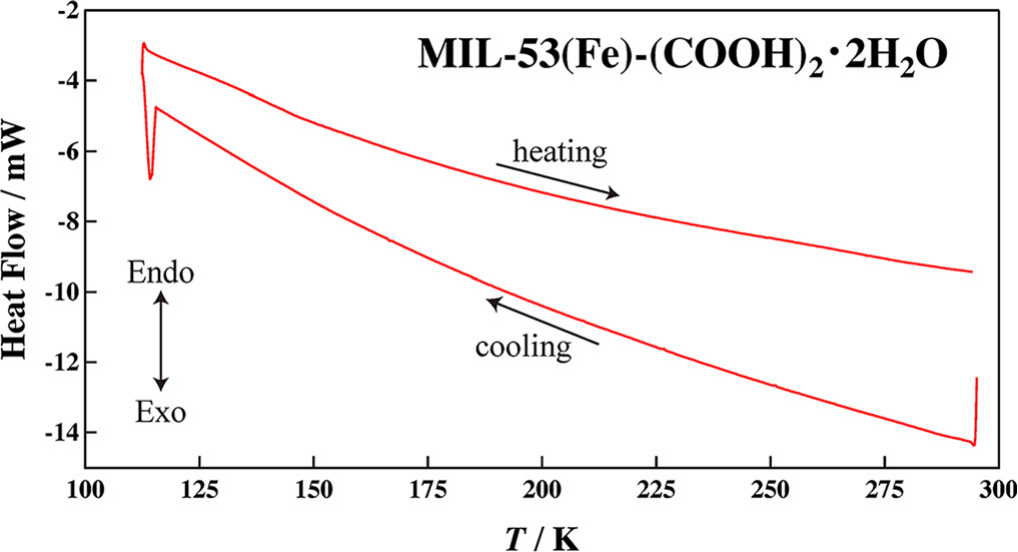
DSC curves of MIL-53(Fe)-(COOH)_2_·2H_2_O.

**FIG. 3.**
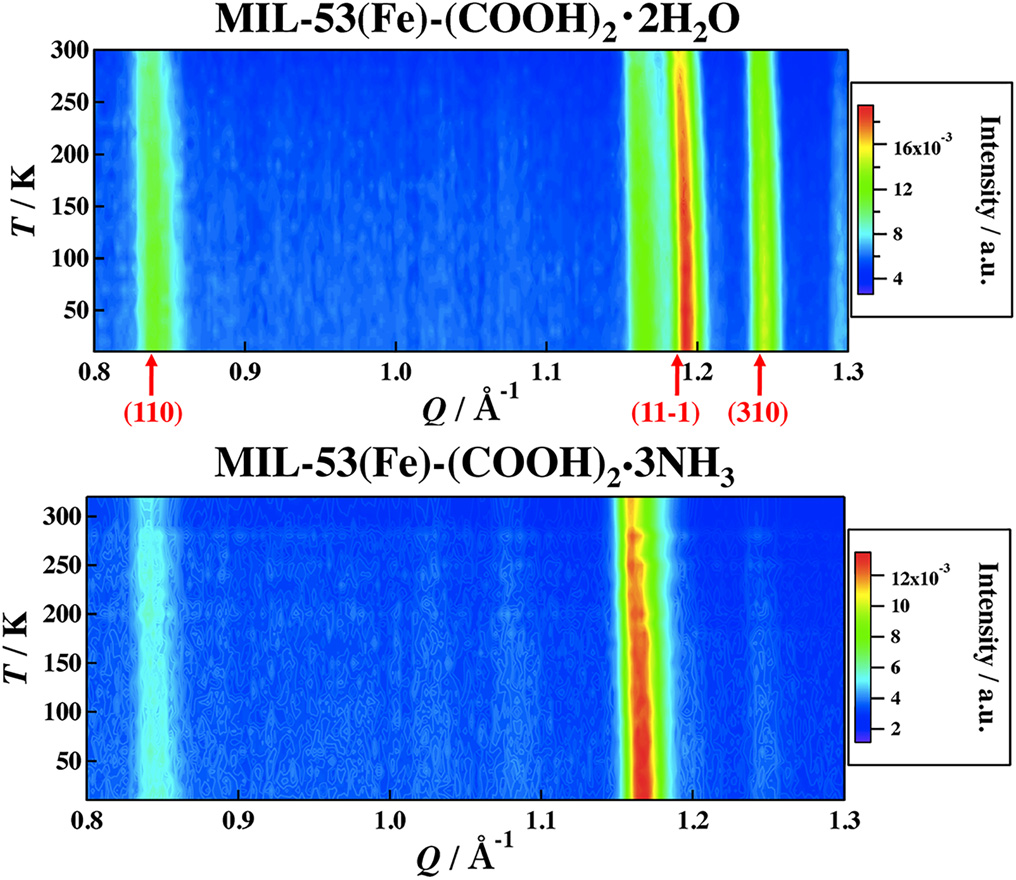
Two-dimensional intensity maps obtained by neutron powder diffraction on TOFTOF. The peak positions calculated from the structure of MIL-53(Fe)-(COOH)_2_·0.88H_2_O^46^ are shown by arrows.

Figure 2 shows the DSC curves of MIL-53(Fe)–(COOH)_2_·2H_2_O in the cooling and heating directions. No thermal anomaly was observed in a temperature range between 110 and 300 K. Figure 3 shows the temperature dependence of the neutron diffraction patterns of MIL-53(Fe)–(COOH)_2_·2H_2_O and MIL-53(Fe)–(COOH)_2_·3NH_3_. As mentioned above, the XRPD patterns of MIL-53(Fe)–(COOH)_2_·1H_2_O and MIL-53(Fe)–(COOH)_2_·3NH_3_ resemble each other and their peak positions are mostly reproduced by the structure of MIL-53(Fe)–(COOH)_2_·0.88H_2_O. In the NPD pattern of MIL-53(Fe)–(COOH)_2_·2H_2_O, however, the (11–1) peak splits into two, implying that the crystal symmetry of MIL-53(Fe)–(COOH)_2_·2H_2_O is lower than that of MIL-53(Fe)–(COOH)_2_·0.88H_2_O. As for temperature dependence, there is no discontinuity of the peak position and intensity in both samples. These results indicate that MIL-53(Fe)–(COOH)_2_·2H_2_O and MIL-53(Fe)–(COOH)_2_·3NH_3_ have no phase transition in contrast to MIL-53(Fe) ·2H_2_O.^46,47^ This may be because the COOH groups placed in the channels contribute to stabilize the channels and suppress the phase transition with the deformation of the channels.

### B. Mean square displacement (MSD)

**FIG. 4.**
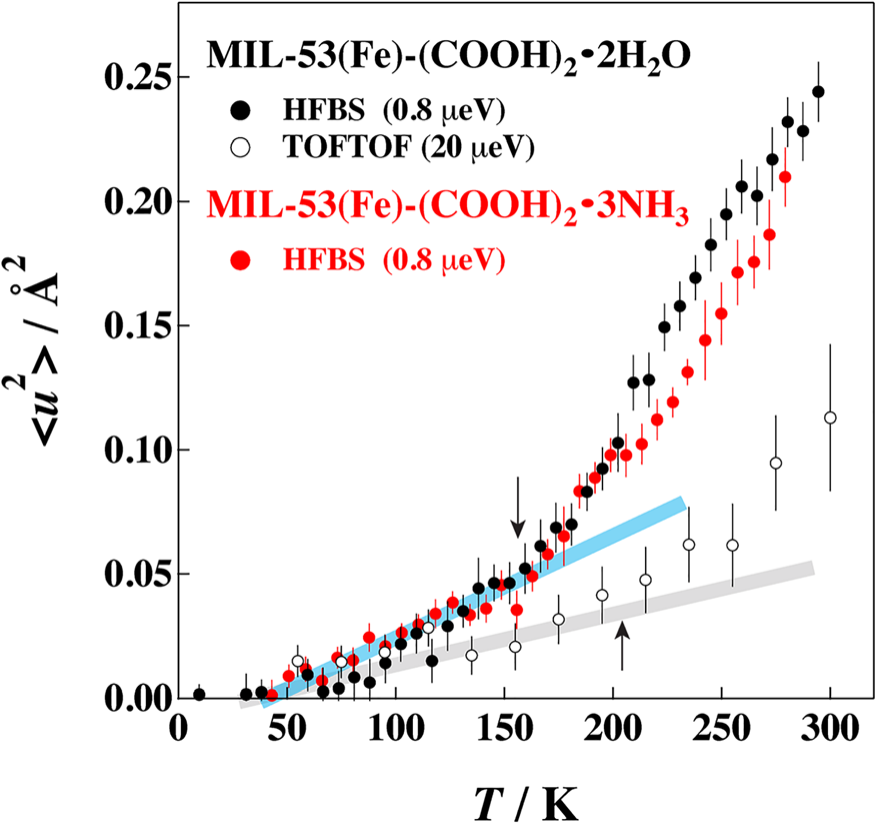
Mean square displacements of MIL-53(Fe)-(COOH)_2_·2H_2_O (black symbols) and MIL-53(Fe)-(COOH)_2_·3NH_3_ (red symbols) as functions of temperature. The closed and open circles correspond to the data taken on HFBS and TOFTOF, respectively. The number in the parenthesis denotes the energy resolution of each instrument. Error bars throughout the text represent one standard deviation.

Figure 4 presents the mean square displacement (MSD) calculated from the intensity data of the fixed window scan assuming the following equation:

$$I\left(Q,t\right)\propto \;\text{exp}\;\left[-\frac{1}{3}Q^{2}\left\langle u^{2}\right\rangle \right].$$ (1)

If all of the vibrational modes are harmonic and there is no relaxation mode for the energy resolution (timescale) of the instrument, MSD is proportional to temperature. As shown here, for both H_2_O and NH_3_ samples, a deviation from a straight line occurred at ca. 150 K in the HFBS data and at ca. 200 K in the TOFTOF data as shown by arrows in Fig. 4. These results indicate that some relaxation modes are activated at around these temperatures. It is reasonable that the MSD data on HFBS with a higher energy resolution exhibit a lower onset temperature than those on TOFTOF. We have measured the QENS data above these temperatures.

### C. Quasielastic neutron scattering

**FIG. 5.**
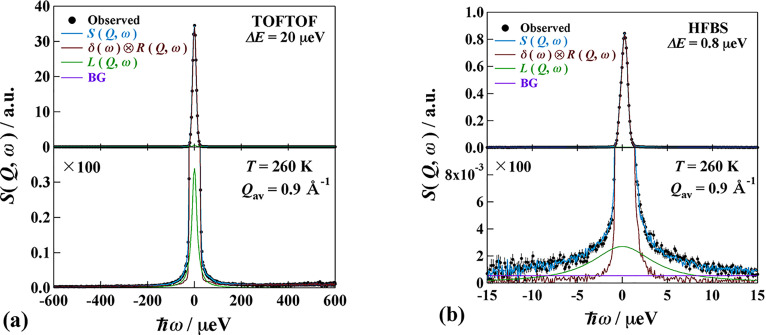
QENS profiles of MIL-53(Fe)-(COOH)_2_·2H_2_O obtained by (a) TOFTOF and (b) HFBS spectrometers. Both data are taken at *T *=* *260 K. The lower panels display the enlarged plot in the vertical axis. See text for the details.

**FIG. 6.**
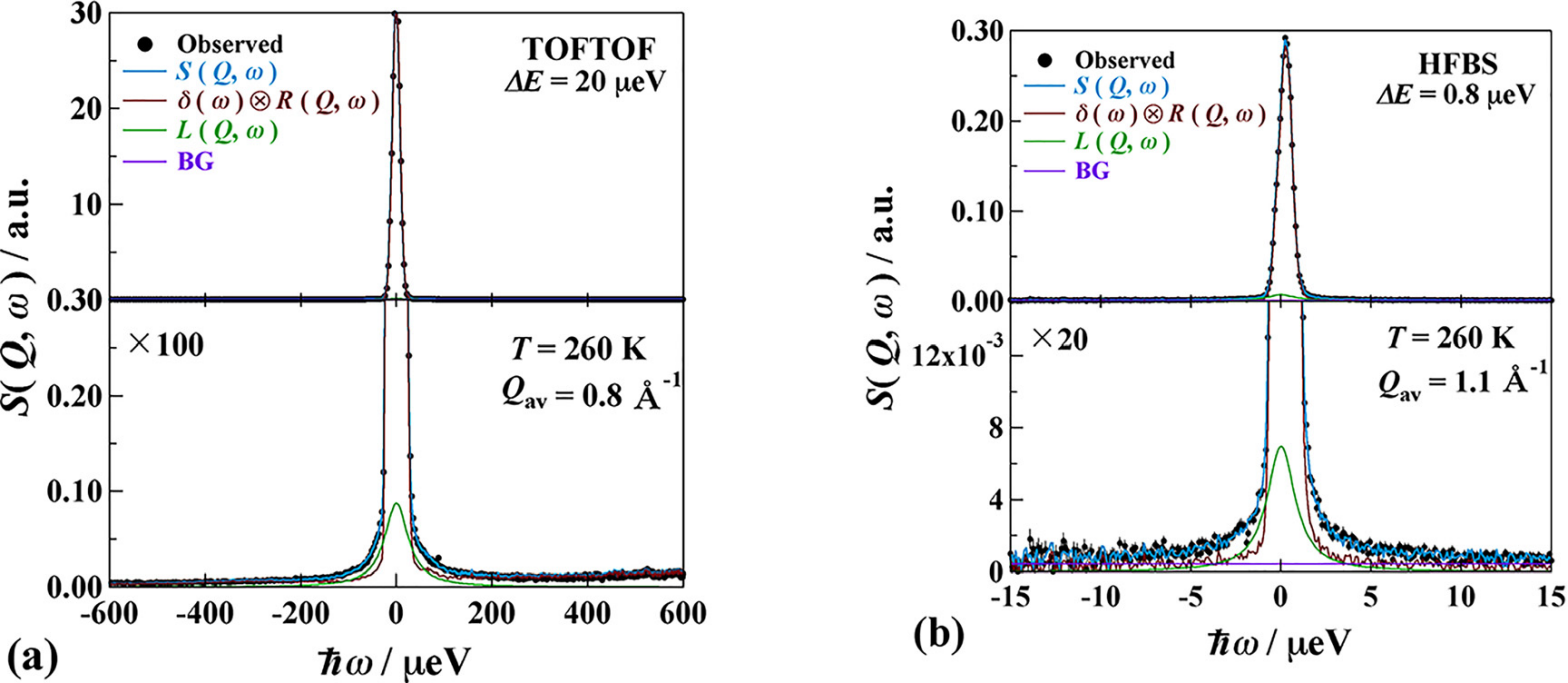
QENS profiles of MIL-53(Fe)-(COOH)_2_·3NH_3_ obtained by (a) TOFTOF and (b) HFBS spectrometers. Both data are taken at *T *=* *260 K. See text for the details.

The QENS spectra (dynamic structure factor) at *T *=* *260 K obtained by TOFTOF and HFBS are demonstrated in Fig. 5 for MIL-53(Fe)–(COOH)_2_·2H_2_O and in Fig. 6 for MIL-53(Fe)–(COOH)_2_·3NH_3_. The bottom figures are expanded vertically for the sake of clarity. As shown here the QENS components are very small (smaller than 10% of an elastic peak). The data of different detectors are summed up to improve the counting statistics as follows: MIL-53(Fe)–(COOH)_2_·2H_2_O; 0.6 Å^−1^ < *Q *<* *1.2 Å^−1^ (*Q*_av_ = 0.9 Å^−1^) for both spectrometers. MIL-53(Fe)–(COOH)_2_·3NH_3_; 0.4 Å^−1^ < *Q* <* *1.2 Å^−1^ (*Q*_av_ = 0.8 Å^−1^) for TOFTOF, 0.5 Å^−1^ < *Q *<* *1.6 Å^−1^ (*Q*_av_ = 1.1 Å^−1^) for HFBS.

The QENS data were fitted by the following equations:

$$S\left(Q,\omega \right)=R\left(Q,\omega \right)⊗\left[A_{\delta }\delta \left(\omega \right)+A_{L}L\left(Q,\omega \right)\right]+\text{BG},$$ (2)

$$L\left(Q,\omega \right)=\frac{1}{\pi }\frac{\Gamma }{{\left(\hbar \omega \right)}^{2}+\Gamma^{2}}.$$ (3)

Here, *R*(*Q*,*ω*) is the resolution function of the instrument and ⊗ is a convolution operator. *δ*(*ω*) is a delta function corresponding to an elastic peak and *L*(*Q*,*ω*) is a Lorentz function with a half width at half maximum (HWHM) *Γ*. *A_δ_* and *A_L_* denote the areas of the delta and Lorentzian components, respectively. BG is a constant background. The fitting was satisfactory for all data as shown by the blue lines in the figures. It is a significant outcome to find the relaxational motion of the NH_3_ molecules carrying protons for the first time.

### D. *Q* dependence of HWHM

**FIG. 7.**
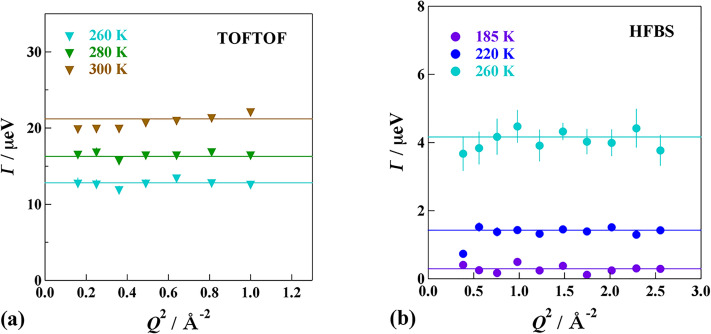
*Q*-dependence of the HWHM estimated from the data of MIL-53(Fe)-(COOH)_2_·2H_2_O taken on (a) TOFTOF and (b) HFBS spectrometers.

**FIG. 8.**
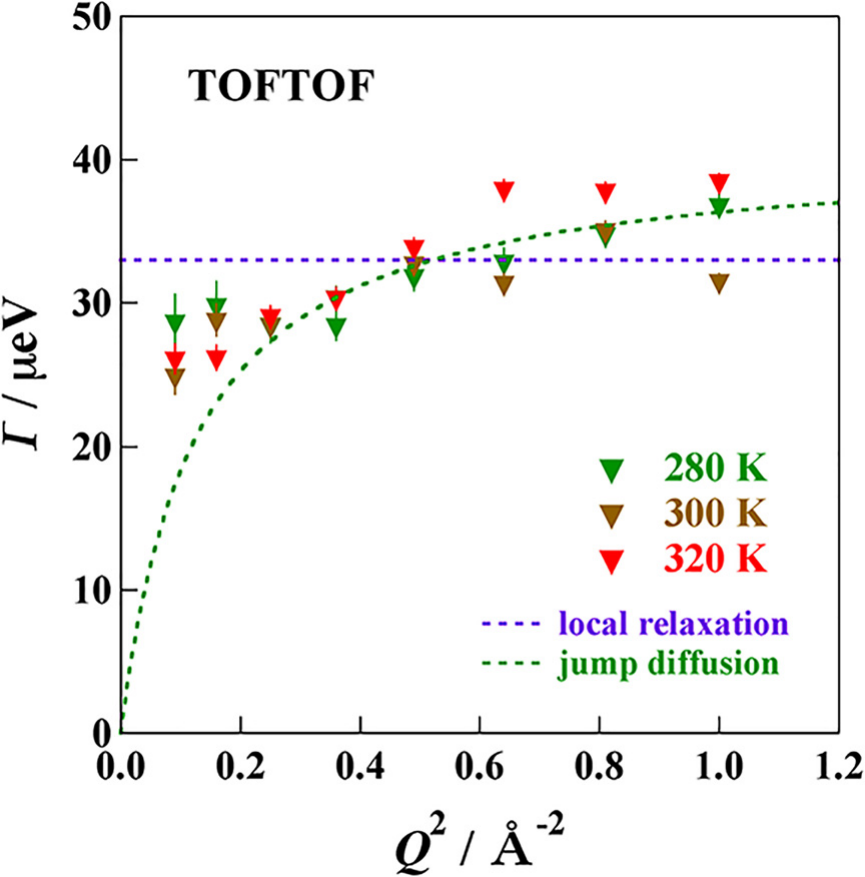
*Q*-dependence of the HWHM estimated from the data of MIL-53(Fe)-(COOH)_2_·3NH_3_ taken on TOFTOF.

Figures 7 and 8 show the *Q*-dependence of the HWHM of the Lorentz function. In these cases, the TOFTOF data are summed up at every 10° and HFBS data of 16 detectors are not summed up. The HWHM has the spatial information of the relaxation observed by the QENS.^59^ If the relaxation is a continuous diffusion such as Brownian motion, the HFHM is given by

$$\Gamma =DQ^{2},$$ (4)

where *D* is a diffusion coefficient. If the relaxation is successive motions of a residence at one site and a jump to another site, which is the most popular diffusion in liquids, the HWHM is represented by

$$\Gamma =\frac{DQ^{2}}{1+{DQ}^{2}\tau_{0}},$$ (5)

where *τ*_0_ is a residence time.^60^ If the relaxation is of a local origin such as a jump between neighboring two sites, the HWHM has no *Q* dependence and given by

$$\Gamma =\frac{1}{\tau_{0}}.$$ (6)

For MIL-53(Fe)–(COOH)_2_·2H_2_O, all the HWHM data measured at different temperatures on both HFBS and TOFTOF exhibit no *Q*-dependence, indicating that the relaxation is of a local mode. For MIL-53(Fe)–(COOH)_2_·3NH_3_, the HWHM Γ seems to increase with an increase in *Q*, though the data quality is not enough, suggesting that the motion of the accommodated NH_3_ molecules has more translational nature. This is consistent with the fact that the activation energy of the NH_3_ compound is smaller than that of the H_2_O compound.

### E. Arrhenius plot

**FIG. 9.**
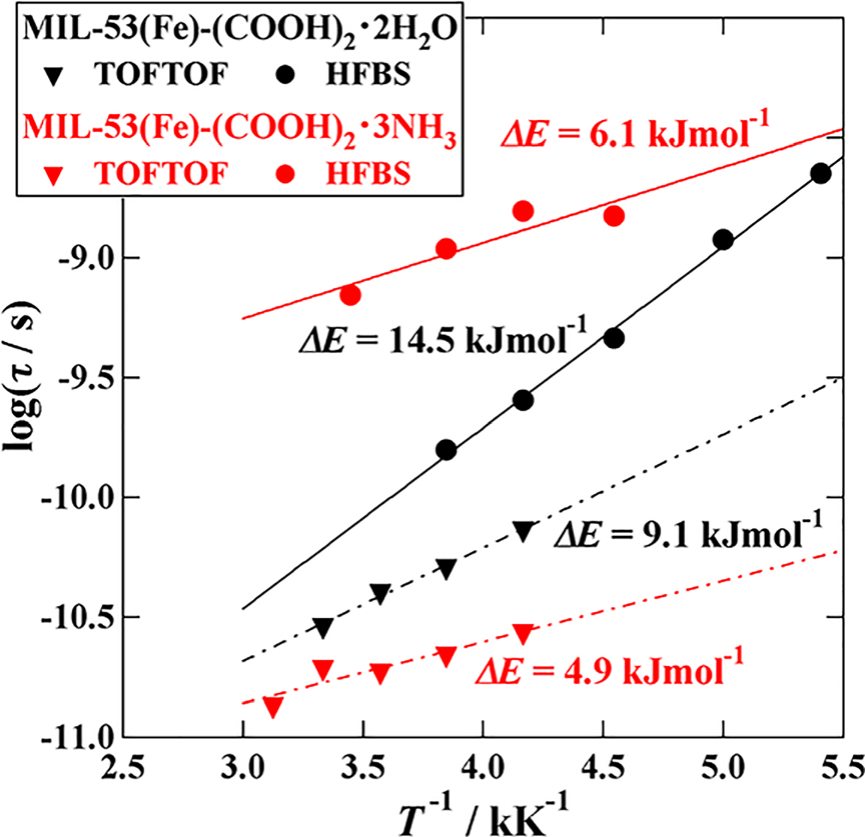
Arrhenius plot for the relaxation times of MIL-53(Fe)-(COOH)_2_·2H_2_O (black symbols) and MIL-53(Fe)-(COOH)_2_·3NH_3_ (red symbols). The circles and triangles represent the data measured on HFBS and TOFTOF, respectively.

Figure 9 is the Arrhenius plot of the relaxation times obtained on the basis of Eq. (6). The *Q* regions for the summation of the *S*(*Q*,*ω*) data are the same as given in Sec. III C. The black symbols denote the data of the H_2_O sample, while the red ones of the HN_3_ samples. For both samples, there are two relaxations; the slower one was observed on HFBS while the faster one on TOFTOF. The activation energy *ΔE* was estimated by fitting the data to the Arrhenius equation,

$$\text{log}\;\tau =\text{log}\;\tau_{0}+\frac{\Delta E}{RT},$$ (7)

where *τ*_0_ is the high-temperature limit of the relaxation time. The obtained *ΔE* values, which are shown in Fig. 9, are all smaller than those in the bulk liquids of H_2_O (17 kJ mol^−1^) and NH_3_ (8.7 kJ mol^−1^).^40,61^ This implies that the intermolecular hydrogen bonds in the channels are weaker than those in the bulk states. This is similar to the cases of our previous MOF-type proton conductors, H_2_dtoaCu·3H_2_O and (NH_4_)_2_(adp)[Zn_2_(ox)_3_]·3H_2_O.^40–42^ It is noted that the relaxation times for the faster relaxations of the H_2_O and NH_3_ compounds are almost the same around 160 K. This is consistent with the fact that the offset temperatures of the excess MSD are almost the same in the H_2_O and NH_3_ compounds (see Fig. 4) even though their activation energies are quite different.

### F. Relaxation intensity

**FIG. 10.**
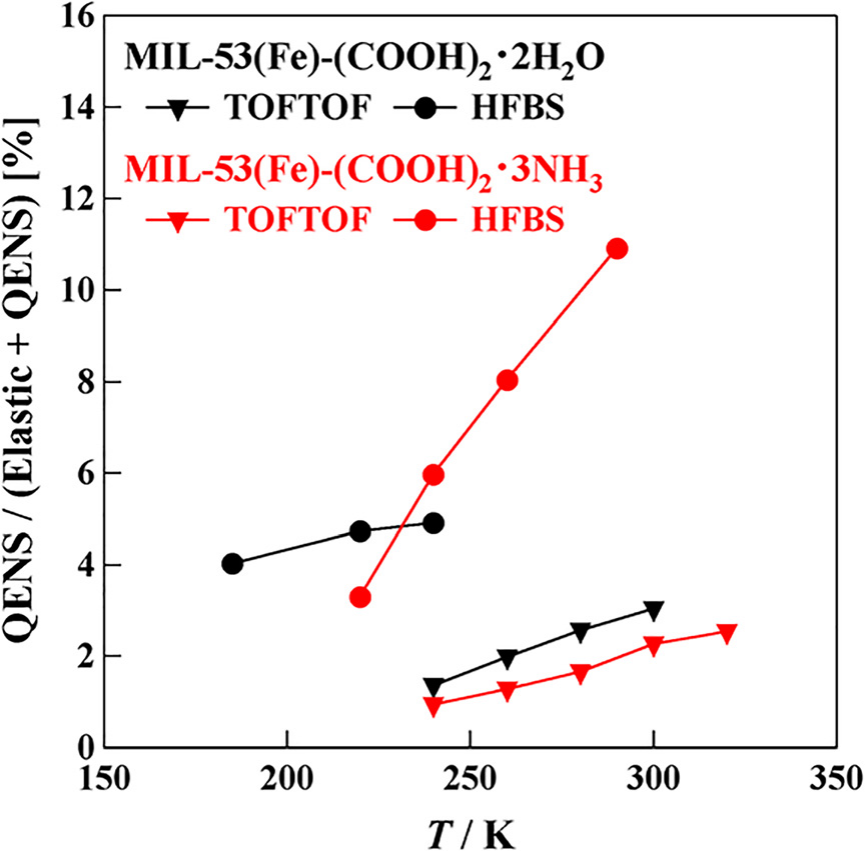
Temperature dependence of the quasi-elastic fractions for the relaxation modes of MIL-53(Fe)-(COOH)_2_·2H_2_O (black symbols) and MIL-53(Fe)-(COOH)_2_·3NH_3_ (red symbols). The circles and triangles represent the data measured on HFBS and TOFTOF, respectively.

Figure 10 shows the quasi-elastic fractions *A_L_*/[*A_L_* +*A_δ_*], where *A_L_* and *A_δ_* are determined in the fitting by use of Eq. (2). In the H_2_O sample, if all of the H atoms of the H_2_O molecules and COOH groups contribute to the relaxations, the fraction is expected to be 66%, but the actual sum of the two relaxation is 7%. In the NH_3_ sample, the expected value is 78% while the experimental one is 11%. Thus, the fractions of the experimental QENS components are much smaller than expected. It should be noted that all of the QENS fractions tend to increase with increasing temperature.

### G. Mechanism of proton conduction

In the proton conduction process, H_2_O and NH_3_ molecules should be the carriers of protons. From the *Q* dependence of the HWHM, the proton conduction is not due to the vehicle mechanism with diffusion of carrier molecules but the Grotthuss mechanism with local rotations of the carrier molecules. The Grotthuss mechanism with NH_3_ molecules is not common but should be possible since an ammonium ion NH_4_^+^ is as stable as a hydronium ion H_3_O^+^ and NH_3_ is a popular hydrogen-bonding liquid as H_2_O.

**FIG. 11.**
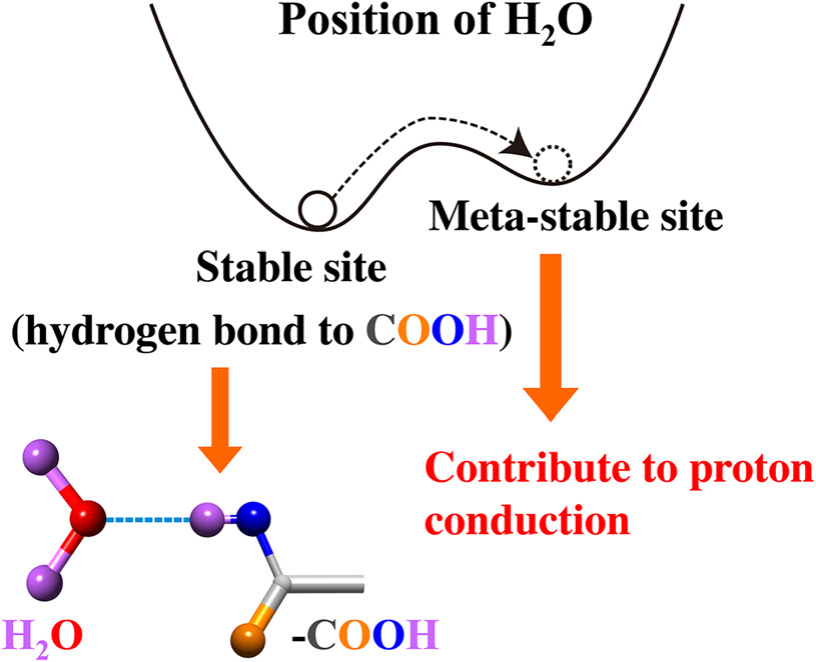
A possible model for the relaxation observed in QENS and related to the proton conduction.

The fractions of the relaxations observed in the QENS measurement are quite small and increase with increasing temperature. This information indicates that the relaxations are originated from the H_2_O and NH_3_ molecules in “excited states.” One possible model (for the H_2_O carriers), which can explain the experimental results, is schematically shown in Fig. 11. In the ground state, H_2_O molecules are hydrogen-bonded to the COOH groups on the surface of the channels. The rotational motions of these H_2_O molecules are too slow to be detected by the QENS method. At higher temperatures, some of the H_2_O molecules are thermally excited and dissociate from the COOH groups. These excited H_2_O molecules or sometimes H_3_O^+^ ions can move much faster as observed by the QENS method and contribute to the proton conduction through the Grotthuss mechanism.

H_2_O molecules can be trapped additionally by the OH groups coordinated to Fe^3+^ ions with hydrogen bonds even though the number of the OH groups is a half of that of the COOH group. This effect is consistent with the fact that two relaxations were found in the QENS experiment. The proton conductivity as a function of temperature is needed for further discussion on the proton conduction mechanism in MIL-53 systems. If the above model is valid, the proton conductivity *σ* should be reproduced with

$$\sigma \propto A_{L1}\text{exp}\left(\frac{\Delta E_{1}}{RT}\right)+A_{L2}\text{exp}\left(\frac{\Delta E_{2}}{RT}\right),$$ (8)

where *A*_L_ and Δ*E* are the fraction of the Lorentz function and the activation energy of each component (1 or 2), respectively [see Eqs. (2) and (7)].

Similar discussion is possible also for the NH_3_ molecules accommodated in MIL-53(Fe)–(COOH)_2_. NH_3_ molecules can be connected to the OH group more preferentially than H_2_O molecules since the OH⋯:N hydrogen bond is energetically more stable than the OH⋯:O hydrogen bond; i.e., *E*(OH⋯:O) = 21 kJ mol^−1^, *E*(OH⋯:N) = 29 kJ mol^−1^.^62^ This may be related to the fact that the mole fraction of NH_3_ accommodated in MIL-53(Fe)–(COOH)_2_ is 3, while that of H_2_O is 2. As for the origin of the two relaxations, however, intra-molecular explanation is also possible; e.g., a rotation about the C_2_ (for H_2_O) or C_3_ (For NH_3_) axis and another 180° rotation with dipole flipping. For further discussion, structural works on the H_2_O and NH_3_ molecules in the channels are essential. Computational approach, such as molecular dynamics simulations, will also be useful.

## IV. CONCLUSION

The quasi-elastic neutron scattering (QENS) of MIL-53(Fe)–(COOH)_2_·2H_2_O and MIL-53(Fe)–(COOH)_2_·3NH_3_ was measured in a wide temperature (*T *=* *6–300 K) and relaxation time (*τ* = 10^−12^–10^−8^ s) ranges. In both samples, two relaxations with Arrhenius temperature dependence are found and their activation energies are smaller than those of bulk H_2_O and NH_3_ liquids. From the momentum transfer dependence of the relaxation time and the temperature dependence of the relaxation intensity, it is can be concluded that the H_2_O and NH_3_ molecules, which are thermally excited and trapped in metastable states, contribute to proton transfer through the Grotthuss mechanism. It is significant to give insight into the proton transfer mechanism with NH_3_ carriers for the first time. More detailed discussion will be possible by adding information from other experiments [e.g., nuclear magnetic resonance (NMR)] and MD simulations.

## Data Availability

The data that support the findings of this study are available from the corresponding author upon reasonable request.
